# Colorectal cancer and screening awareness and sources of information in the Hungarian population

**DOI:** 10.1186/s12875-018-0799-1

**Published:** 2018-06-30

**Authors:** Noémi Gede, Diána Reményi Kiss, István Kiss

**Affiliations:** 1 Institute for Translational Medicine, Medical School, and Doctoral School of Health Sciences, Faculty of Health Sciences, University of Pécs, Szigeti út 12, Pécs, 7624 Hungary; 2 Doctoral School of Health Sciences, University of Pécs, Pécs, Hungary; 3 Department of Public Health Medicine, Medical School, University of Pécs, Pécs, Hungary

**Keywords:** Colorectal cancer, Cross-sectional, Awareness, Screening, Prevention

## Abstract

**Background:**

This study aims to survey the level of awareness of colorectal cancer and screening and to identify sources of information among the population under investigation.

**Methods:**

A cross-sectional study was conducted among 1150 adults between the ages of 40 and 70 using quota sampling. Data were collected through self-made questionnaires to be completed by respondents.

**Results:**

32.7% of the participants correctly identified the recommended beginning of colorectal cancer screening, these participants were more likely to see their physician more frequently in the past years than those answering to the qusetion incorrectly (*p* = 0.008). 22.4% of the respondents were in possession of appropriate information on the frequency of colorectal cancer screening and had a relatively high level of educational attainment (*p* < 0.001). Very few respondents were well-informed about the risk factors and symptoms of colorectal cancer. Those who were well-informed were likely to live in a county town (*p* < 0.001) and to have a relatively high level of educational attainment (*p* < 0.001). They were most likely to have accessed their information on the internet. 27.0% of respondents had not heard of CRC screening methods before. They were likely to be male and relatively young and to have a relatively low level of educational attainment. Furthermore, they saw their doctor relatively seldom. The respondents who had heard about screening methods were most likely to have gathered their information from health workers. Conclusions: The majority of respondents did not have sufficient information about colorectal cancer and screening. This is particularly true of less educated, younger male participants who do not live in a county town and of respondents who see their physician relatively seldom. Sources of information should be used more effectively, thus yielding an increased level of awareness.

**Electronic supplementary material:**

The online version of this article (10.1186/s12875-018-0799-1) contains supplementary material, which is available to authorized users.

## Background

Colorectal cancer (CRC) was the fourth leading cause of cancer death worldwide in 2012. Incidence of CRC was more than 8500 in Hungary in 2012, and mortality was nearly 4500 [1]. In 2013, from among the 28 member states of the European Union, the highest standardised death rate could be found in Hungary, followed by Slovakia [2]. In Hungary, CRC is most commonly recognized at a late stage, when options for curative care are limited [3].

CRC has several risk factors, of which the most important are the following: obesity, a sedentary lifestyle, smoking, excessive consumption of alcohol and red meats, a low-fibre, high-fat diet, a positive family history and age (over 50) [4–6].

The aims of screening are to reduce the risk of death from CRC through early detection and the occurrence of complications associated with detection of cancer at a later stage. Such screening also purposes to decrease the incidence and mortality of CRC by detection and removal of precancerous lesions [7]. According to a meta-analysis, faecal occult blood testing (FOBT) and sigmoidoscopy screening yielded a reduction of 18 and 26% in CRC mortality, and reduction of 8 and 27% in late-stage CRC incidence respectively [8].

The two-stage screening method is used in Hungary in accordance with the recommendations of the Chief Medical Officer of Hungary. In people of average risk, FOBT should be carried out biannually, and, in the case of a positive test result, a colonoscopy should be performed [9]. It should be noted that only opportunistic CRC screening programmes were conducted before 2017. Colorectal screening has been conducted nationwide since then, like cervical and breast cancer screenings. Awareness among laypeople about CRC (i.e., risk factors and symptoms) and CRC screening has proved to be insufficient; only about 21–44% of the participants in the relevant studies exhibited appropriate knowledge of CRC screening [10–14]. Many reports have shown a significant relationship between awareness and willingness to participate. Being well-informed has a positive effect on participation in CRC screening [15–17]. However, participation in CRC screening is very low in Hungary [18]. Low rates can also be observed in various other countries, [19, 20] but efforts are being made to improve the situation [21, 22]. A comprehensive and representative report on people’s awareness has not yet been drafted in Hungary. The lack of such a report may be one of the main reasons for avoidance of CRC screening. This study aims to survey level of awareness and to ascertain sources of information among the population under investigation.

## Methods

The study was conducted in 23 general practitioner districts in Baranya County in southwestern Hungary from 2015 to 2016. Surveys were administered within a few weeks in each site of sampling. Sites collected the data within three working days per week, the proportion of morning and afternoon office hours was 1 to 1.

### Participants

1150 people between the ages of 40 and 70 were recruited using quota sampling. Participants were included by observing the distribution by sex and place of residance reported in the database of Hungarian Central Statistical Office. The exclusion criterion was a diagnosed cancerous disease. 138 people were excluded because of a lack of responses. Consequently, data from 1012 participants (88.0%) were evaluated. Data were collected through self-made questionnaires to be completed by respondents in the waiting room of their general practitioner. The questionnaires and written informed consent forms were distributed by assistants in the waiting room and proper time was given to decide whether to participate in the study. All participants remained anonymous in this study. Ethical approval was received from the Regional and Institutional Ethical Committee of the University of Pecs, Hungary.

### Questionnaire survey

Age, sex, place of residence, educational attainment, financial situation and religiosity were surveyed. The respondents assessed their financial situation on a Likert scale ranging from very poor (=1) to very good (=5).

Similarly, they answered certain multiple-choice questions, such as recommended beginning, frequency and protocol of CRC screening, and importance of early detection and asymptomatic development of CRC. The participants assessed their knowledge of CRC screening, and at the end of the questionnaire they were provided the opportunity to decide whether they wished to receive more information. The study surveyed knowledge of CRC screening methods, risk factors and symptoms, and sources of information. Participants were able to provide multiple responses to all these questions. The responses to some of the questions were reduced to dichotomous variables for the sake of extensive analysis. Respondents who chose six correct symptoms and no more than one incorrect symptom or five correct symptoms without incorrect symptoms were regarded as well-informed, and all other respondents were evaluated as not being well-informed. Respondents who chose eight correct risk factors and no more than one incorrect risk factor or seven correct symptoms without incorrect symptoms were regarded as well-informed, and everybody else was seen as not being well-informed (Additional file 1).

### Statistical analyses

The analysis was performed with descriptive statistics – mean, median and relative frequency – a goodness-of-fit χ2 test, binominal and one-sample median tests, odds ratio, the Kruskal–Wallis test with the Mann–Whitney test as a post hoc test and the Bonferroni correction. A two-sided *p* value of < 0.05 was regarded as statistically significant.

The available-case analysis was used for missing data. Statistical analyses were performed with SAS software version 9.2 (SAS Institute Inc., Cary, NC).

## Results

### Sample characteristics

The socio-demographic characteristics of the sample are provided in Table 1. The data for the population under investigation were compared to data for Baranya County at the Hungarian Central Statistical Office. Based on the comparison, the sample is representative with respect to age (*p* = 0.462), sex (*p* = 0.745) and place of residence (*p* = 0.846). 0.9% of the respondents had received less than a primary school education, 11.8% had completed primary school, 31.0% had earned a vocational school certificate, 39.1% had a secondary school education, and 17.2% had received a college/university degree. Participants rated their financial situation as follows: very poor (3.8%), poor (17.5%), acceptable (45.4%), good (30.5%) and very good (2.8%). 52.3% of the respondents were religious.

**Table 1:** Socio-demographic characteristics in terms of representativeness

		Population (*N* = 1012)	Hungarian Central Statistical Office (*N* = 159,758)
Age	Mean	54.4	54.4
	Median	55	55
		N (%)	N (%)
Sex	Male	470 (46.4%)	75,086 (47.0%)
	Female	542 (53.6%)	84,672 (53.0%)
Place of residence	County town	395 (39.0%)	61,028 (38.2%)
	Other town	266 (26.3%)	42,176 (26.4%)
	Village	351 (34.7%)	56,554 (35.4%)

### Awareness of guideline and importance of CRC screening

32.7% of the participants knew the recommended beginning of colorectal cancer screening. 22.4% of the respondents provided correct answers about the required frequency of CRC screening, 59.2%, knew the CRC screening protocol, 69.6% were aware that early-stage CRC is a curable disease, and 56.2% understood that there is an asymptomatic period in the development of CRC. Participants who knew the recommended beginning of colorectal cancer screening were more likely to see their physician more frequently in the past years than those answering to the qusetion incorrectly (*p* = 0.008). Patients providing a correct answer about required frequency, protocol, curability of early stage CRC, and asymptomatic period of CRC had significantly higher levels of educational attainment as compared to those providing a wrong answer (*p* < 0.001, *p* < 0.001, *p* = 0.009 and *p* < 0.001, respectively).

### Awareness of CRC screening methods

**Fig. 1:**
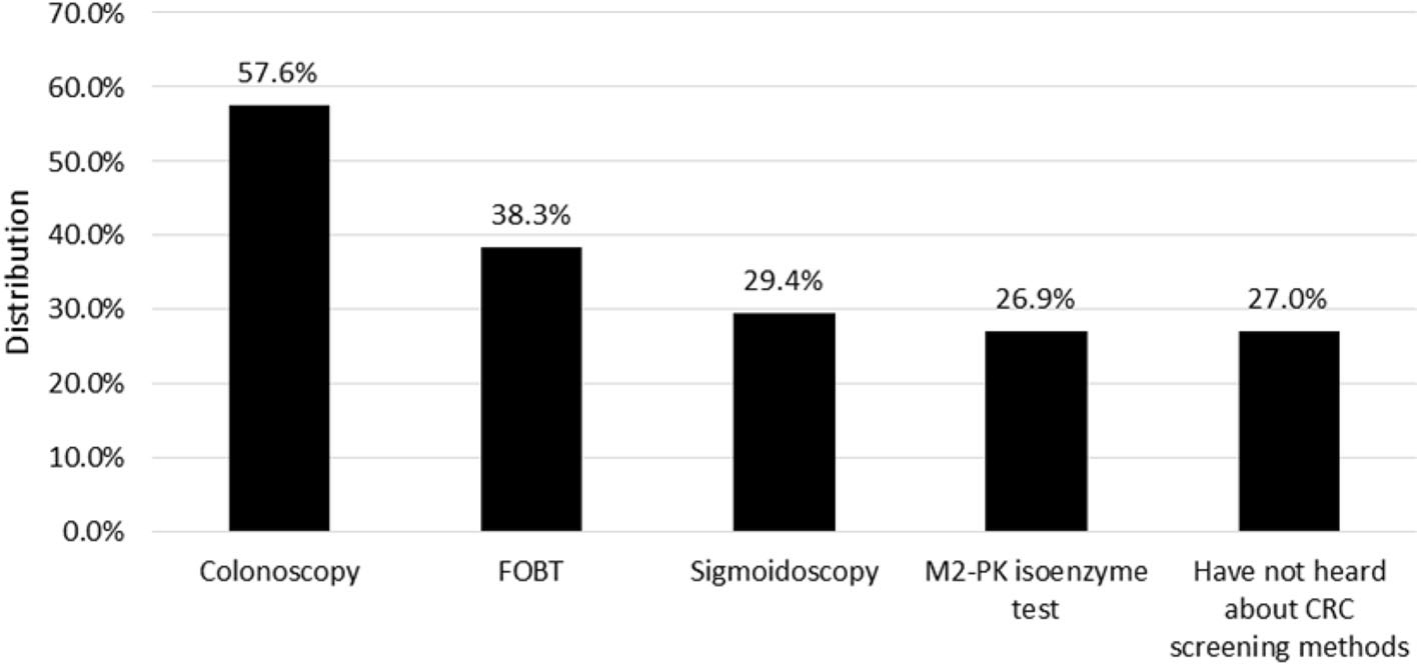
Awareness of CRC screening methods. CRC: Colorectal Cancer FOBT: Faecal Occult Blood Test M2-PK: pyruvate kinase isoenzyme type M2

The respondents indicated which screening methods they had heard of. These distributions are provided in percentage form in Fig. 1. Those who had not heard about CRC screening methods were likely to be male (OR = 1.71; 95% CI: 1.29–2.26) and relatively young (*p* = 0.002); they had a relatively low level of educational attainment (*p* < 0.001) and saw their physician relatively seldom (*p* < 0.001).

### Awareness of CRC risk factors and symptoms

18.8% of the respondents were well-informed about the risk factors of CRC, and 81.2% were not. 21.0% were well-informed about the symptoms of CRC, and 79.0% were not. Those who were well-informed were likely to live in a county town (*p* < 0.001) and have a relatively high level of educational attainment (*p* < 0.001). 14.1% had a positive family history for CRC, but only 53.0% of those considered family history a risk factor.

### Sources of information about CRC

**Fig. 2:**
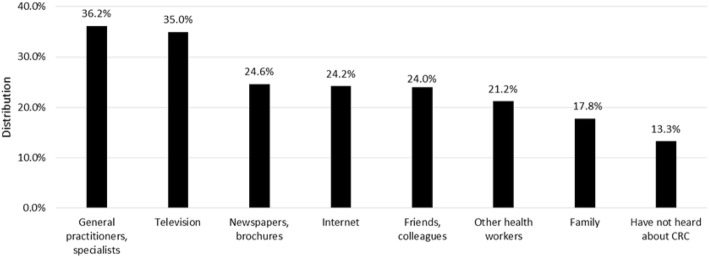
Sources of information about CRC. CRC: Colorectal Cancer

The participants indicated their source of information about CRC. These distributions are provided in percentage form in Fig. 2. There is a significant relationship between the sources of information indicated and knowledge about CRC and screening; socio-economic characteristics are provided in Tables 2 and 3. Patients who had never heard about CRC saw their physician less frequently (*p* < 0.001).

**Table 2:** Relationship between source of information and socio-economic characteristics

Source of information	Socioeconomic characteristics	*P* value
General practitioners, specialists	Users are older	*p* < 0.001
Internet	Users are younger	*p* < 0.001
	Users have better financial situation	*p* = 0.001
Newspapers, brochures	Users are older	*p* = 0.046
I have never heard about CRC	Users are younger	*p* = 0.010

A user is a person who answered YES to the question about using that specific source of information in the questionnaire

**Table 3:** Relationship between source of information and awareness of CRC and screening

Source of information	Information about CRC and screening	odds ratio
General practitioners, specialists	Users were likely to have heard about FOBT	OR = 3.61; 95% CI: 2.76–4.73
	Users were likely to have heard about the M2-PK isoenzyme test	OR = 2.32; 95% CI: 1.74–3.08
	Users were likely to have heard about colonoscopy	OR = 2.77; 95% CI: 2.10–3.66
Other health workers	Users were likely to have heard about FOBT	OR = 3.60; 95% CI: 2.63–4.93
	Users were likely to have heard about the M2-PK isoenzyme test	OR = 2.49; 95% CI: 1.81–3.42
	Users were likely to have heard about colonoscopy	OR = 2.41; 95% CI: 1.72–3.36
Internet	Users were likely to be well-informed about risk factors	OR = 3.32; 95% CI: 2.38–4.64
	Users were likely to be well-informed about symptoms	OR = 2.63; 95% CI: 1.90–3.63
	Users were likely to have heard about sigmoidoscopy	OR = 2.02; 95% CI: 1.50–2.74

A user is a person who answered YES to the question about using that specific source of information in the questionnaire

### Judgement of the level of awareness

26.0% of the population under investigation thought that their knowledge about CRC screening was appropriate. They were less likely to have a relatively high level of educational attainment (*p* < 0.001), and they were females (OR = 1.52; 95% CI: 1.14–2.02). 67.4% wanted to obtain more information about CRC screening. These respondents were likely to be religious (OR = 1.52; 95% CI: 1.17–1.99) and they had not heard of CRC screening before (OR = 1.68; 95% CI: 1.26–2.25).

## Discussion

32.7% of the participants knew the recommended beginning of colorectal cancer screening. In similar cross-sectional studies, this rate was more favourable (47.9%; 83.0%) [23, 24]. These respondents were likely to see their physician more frequently. Furthermore, 22.4% of the participants knew the frequency of CRC, 59.2% had accurate information about protocol, and 56.2% were informed about the development of CRC. 69.6% of the respondents knew the importance of early detection. A previous report, however, showed a better rate (78.5%) [25]. Those people who had suitable information about the frequency and protocol of CRC screening and its importance were likely to have a relatively high level of educational attainment.

The most frequently mentioned screening methods were colonoscopy and FOBT. 27.0% of the participants had not heard about CRC screening methods before. Berkowitz et al. also surveyed respondents who had not heard of CRC screening methods, and, compared to our study, their rate was higher (42.0%) [26]. They were likely to be relatively young males who had a relatively low level of educational attainment and saw their physician relatively seldom.

81.2% of the respondents were not well-informed about the risk factors. In a previous study, this rate was more than 90.0% [27]. Furthermore, merely 53.0% of the participants with a positive family history for CRC considered heredity a risk factor. 79.0% of the respondents were not well-informed about the symptoms. In the study just noted, this rate was over 90.0% [27]. Both studies showed that higher education affects awareness positively, but our study found that the place of residence also had a significant effect on awareness.

Sources of information were as follows: general practitioners or specialists (36.2%), television (35.0%), newspapers and brochures (24.6%), internet (24.2%), friends or colleagues (24.0%), other health workers (21.2%) and family (17.8%). A small proportion of respondents (13.3%) had never heard about CRC before. This rate was higher in a cross-sectional study (22.0%) [28]. In an Italian report, this order was different from the results of our study: (1) friends, (2) television, (3) newspapers, (4) general practitioner and (5) specialists [29]. Older respondents were likely to obtain their information about CRC from general practitioners, specialists, newspapers and brochures. However, younger respondents and participants in a relatively good financial situation were likely to use the internet to gather information. Respondents who were well-informed about risk factors and symptoms were most likely to access their information on the internet. Respondents who had heard about the non-invasive screening method and colonoscopy were most likely to learn about them from general practitioners and other health workers, but in the case of sigmoidoscopy, the internet was the most characteristic source of information. Participants who had not heard about CRC were likely to be younger and see their physician relatively seldom.

Very few respondents considered their knowledge about CRC suitable. They were likely to be females with a relatively high level of educational attainment. However, a significant number of respondents (67.4%) were open to obtaining more information. A previous survey found a nearly similar rate (60.0%) [24]. Religious respondents and people who had not heard about CRC screening were more open to new information.

### Limitations

Cross-sectional surveys do not permit causal generalizations. Another limitation of the study is the nonprobability sampling used, raising concerns about the occurrence of self-selection bias. We attempted to minimise this by collecting a sample that was representative with respect to age, sex and place of residence. It was people who saw their general practitioner who were recruited for this study; this could have caused bias on the following question: “How often do you see your doctor?”. General practitioners and assistants were asked not to aid participants in completing the questionnaires so as not to influence their answers.

## Conclusions

The decisive majority of respondents did not know the CRC screening guideline and did not have accurate information about CRC risk factors and symptoms. Furthermore, a significant number of respondents had not heard about CRC screening methods. This lack of information can result in a low rate of participation in CRC screenings, since adequate knowledge is essential for participation. Most of the respondents were open to new information. To broaden people’s awareness of this topic, health promotion programmes should focus on males, relatively young people, those who have a relatively low level of educational attainment, and those who do not live in a county town and do not see their physician regularly. Health workers and the internet have a significant role in mediating information. Consequently, these sources of information should be strengthened.

## Additional file

Additional file 1: Data were collected through self-made questionnaires. The questionnaire contains 19 questions (dichotomous questions, single-answer multiple choice questions, multiple-answer multiple choice questions, and likert-type scales questions). (DOCX 28 kb)

## Abbreviations

CRC: Colorectal Cancer
FOBT: Faecal Occult Blood Test
M2-PK: pyruvate kinase isoenzyme type M2
