# Supplementary material for: Colorectal cancer and screening awareness and sources of information in the Hungarian population
Source: BMC Fam Pract. 2018 Jun 30;19:106. doi: 10.1186/s12875-018-0799-1 (PMC6026511; doi:10.1186/s12875-018-0799-1)
Supplement: Supplementary file 1 — Data were collected through self-made questionnaires. The questionnaire contains 19 questions (dichotomous questions, single-answer multiple choice questions, multiple-answer multiple choice questions, and likert-type scales questions). (DOCX 28 kb) [file 12875_2018_799_MOESM1_ESM.docx]

1. **Age:** ………… years
2. **Sex:**

O Male

O Female

1. **Where do you live?**

O County town

O Other town

O Village

1. **What is your highest level of education?**

O Less than primary school

O Primary school

O Vocational school / Industrial school

O Secondary school

O College/University

1. **How do you judge your financial situation?**

1. **Are you religious?**

O Yes

O No

1. **How often do you visit your family doctor and specialists?**

O Never

O Every 2-3 year

O Annually
O Once in a half a year
O Quarterly

O Monthly

1. **Have you ever heard about the following screening methods? (You can indicate multiple answers)**

☐ Faecal occult blood test (detection of blood from stool)

☐ M2-PK isoenzyme test (detection of a specific enzyme from stool)
☐ Sigmoidoscopy (endoscopic examination of the lowest part of colon)
☐ Colonoscopy (endoscopic examination of the colon)
☐ I have not heard about any of these screening methods.

1. **What is the recommended beginning of colorectal cancer screening in an average risk population?**

O above the age of 40 years

O above the age of 50 years

O above the age of 60 years

O above the age of 70 years

O I do not know.

1. **How often is the participation in colorectal screening recommended for the average risk population?**

O Every half a year

O Annually

O Biannually

O Every three year

O I do not know.

1. **Which of the followings fit the protocol of colorectal cancer screening in Hungary?**

O ”one-stage”, screening with stool examination.

O ”one-stage”, the screening is performed with colonoscopy.

O ”two-stage”, the first stage is stool examination, in the case of a positive test result, a colonoscopy should be performed in the second stage.

1. **Do you consider yourself well-informed about colorectal cancer screening?**

O Yes

O No

1. **Is an early-stage colorectal cancer curable?**

O Yes

O No

O I do not know.

1. **Does/Did any case of colorectal cancer occur in your family?**

O Yes

O No

O I do not know.

1. **Can an early-stage colorectal cancer be asymptomatic?**

O Yes

O No

O I do not know.

1. **From which sources have you gathered information about colorectal cancer? (You can indicate multiple answers)**

☐ General practitioners, specialists

☐ Other health workers (nurse, assistance, etc.)

☐ Friends, colleagues

☐ Family members

☐ Television

☐ Internet

☐ Newspapers, brochures

☐ Other:…………………………..

☐ I have not heard about colorectal cancer.

1. **Please, choose risk factors contributing to the development of colorectal cancer! (You can indicate multiple answers)**

☐ Low intake of fruit and vegetable

☐ High intake of red and grilled meat

☐ Poultry meat consumption

☐ Alcohol abuse

☐ High fluid intake

☐ High-calorie diet, particularly fat-rich

☐ Positive family history

☐ Age above 50 years

☐ Overweightedness

☐ Inflammatory bowel diseases

☐ Intestinal infections

☐ Smoking

☐ High blood pressure

☐ Sedentary lifestyle

☐ Oral contraceptives (‘pills’)

☐ Use of painkillers

☐ Superficial polyps in the colon

1. **Please, choose the most typical symptoms of colorectal cancer! (You can indicate multiple answers)**

☐ Strong, crampy abdominal pain

☐ Unintentional weight loss

☐ Change in bowel habits, including diarrhea or constipation

☐ Weakness or fatigue

☐ Frequent nausea, vomiting

☐ High blood pressure

☐ Elevated level of blood glucose

☐ Bloating, or a feeling of incomplete emptying of the bowels after having a bowel
 movement

☐ Blood in your stool

☐ Fever

☐ Dizziness

☐ Poor appetite

☐ A lump around the back passage (anus)

☐ Rectal bleeding

1. **Would you like to get more information about colorectal cancer screening?**

O Yes

O No
